# Tracking the soluble biomarkers of MIS-C and their association with clinical parameters and severity

**DOI:** 10.1038/s41390-025-04542-8

**Published:** 2025-10-31

**Authors:** Tetyana Pidkova, Rosa Maria Pino-Ramirez, Victor Urrea, Marta Vidal, Ruth Aguilar, Gemma Moncunill, Jordi Antón, Bonaventura Clotet, Carlota Dobaño, Claudia Fortuny, Julià Blanco, Benjamin Trinité

**Affiliations:** 1 IrsiCaixa, Badalona, Spain; 2 Hospital Sant Joan de Déu, Universitat de Barcelona, Esplugues de Llobregat, Spain; 3 ISGlobal, Barcelona, Spain; 4 CIBER de Enfermedades Infecciosas (CIBERINFEC), Madrid, Spain; 5 Infectious Diseases and Systemic Inflammatory Response in Pediatrics, Pediatric Infectious Diseases Department, Institut de Recerca Sant Joan de Déu, Barcelona, Spain; 6 Centre for Biomedical Network Research on Epidemiology and Public Health (CIBERESP), Madrid, Spain; 7 Departament de Cirurgia i Especialitats Medicoquirúrgiques, Facultat de Medicina i Ciències de la Salut, Universitat de Barcelona, Barcelona, Spain; 8 Germans Trias I Pujol Research Institute (IGTP), Can Ruti Campus, Badalona, Spain; 9 University of Vic-Central University of Catalonia (UVic-UCC), Vic, Spain

## Abstract

**Background:**

Multisystem Inflammatory Syndrome in Children (MIS-C) is a severe post-COVID-19 complication characterized by hyperimmune activation and systemic inflammation. Identifying biomarkers for disease severity and predicting ICU admission is critical.

**Methods:**

This study analyzed 92 inflammatory biomarkers in serum samples from 22 pediatric MIS-C patients and 17 convalescent COVID-19 children using Olink proteomics. MIS-C samples were taken at diagnosis, before treatment, and at follow-up. Convalescent COVID-19 samples were taken 3 to 5 weeks after acute COVID-19 diagnosis, at a similar time to MIS-C theoretical occurrence.

**Results:**

The study identified 29 significantly altered biomarkers in MIS-C, including elevated pro-inflammatory cytokines (IFN-γ, IL-6, TNF, IL-18), chemokines (CXCL9, CXCL10, CXCL11), and immune regulators (IL-10, PD-L1, CDCP1). Among these, random forest analysis highlighted 18 key markers distinguishing MIS-C from convalescent cases. ICU-admitted MIS-C patients displayed distinct biomarker signatures, and the differential regulation of IL-2, IL-33, CD244, SCF, TNFRSF9, and CD8α, was sufficient to predict almost all ICU admissions. Notably, lower levels of soluble CD244 and TNFRSF9 correlated with longer hospital stays. Finally, longitudinal analysis showed biomarker normalization within two months post-MIS-C.

**Conclusion:**

This study provides a detailed characterization of the MIS-C inflammatory response, identifying potential biomarkers for improved early diagnosis and clinical management.

**Impact:**

- MIS-C incidence has substantially decreased since the beginning of the COVID-19 pandemic, but rare cases are still occurring with severe to critical presentation.
- We identified 6 biomarkers, IL-2, IL-33, CD244, SCF, TNFRSF9, and CD8α, predictors of ICU-admission in MIS-C.
- We showed the normalization of cytokine profiles by 2 months following treatment in MIS-C.
- This study Identified potential biomarkers for improved early diagnosis and clinical management of MIS-C.

## Introduction

COVID-19 generally affects children less severely, with most cases being asymptomatic or presenting with mild symptoms such as fever and cough,^1–4^ however, some children can develop more severe disease and require hospitalization,^5^ especially children with underlying medical conditions.^6^ An additional concern has been post-infectious immune disorders, and among these, multisystem inflammatory syndrome in children (MIS-C), which has emerged as a rare but significant COVID-19-associated complication first described in Spring 2020.^7^ It is defined by persistent fever, multisystem involvement, elevated inflammatory markers, and past SARS-CoV-2 exposure.^4,8^ Symptoms typically happen 2 to 6 weeks after acute SARS-CoV-2 infection, though in rare cases, symptoms have been reported more than six weeks after infection.^9^ MIS-C is driven by a hyperimmune response, causing inflammation that affects multiple organs, with at least two organ systems.^10,11^ While MIS-C is considered a severe condition, about half of the patients undergo intensive care unit (ICU) admission because of more critical symptoms such as cardiac involvement (elevated troponin or N-terminal prohormone of brain natriuretic peptide/NT-proBNP), hemodynamic instability (shock or arrhythmia), or respiratory distress, and key parameters such as elevated inflammatory markers (D-dimer, C-reactive protein/CRP).^10,12–14^ Children in general inpatient care exhibit more stable vitals and less multi-organ involvement. Treatments include intravenous immunoglobulin (IVIG) with steroids, and interleukin IL-6 or IL-1 inhibitors for unresponsive cases.^15,16^ Anticoagulation therapy is recommended to prevent thrombosis, with supportive care like fluids, antibiotics, and, in severe cases, ventilation or vasopressors.^14,17,18^ Longitudinal studies show that systemic markers often normalize within 2–4 weeks after treatment, with most patients showing full inflammatory and cardiac recovery by 3–6 months.^19–21^

Clinically, MIS-C shares features with Kawasaki disease, toxic shock syndrome, and macrophage activation syndrome—including fever, mucocutaneous inflammation, cardiovascular dysfunction, and cytokine elevation—which complicates differential diagnosis.^22–24^ Previous MIS-C studies identified elevated biomarkers associated with type II interferon (IFN) signaling (IFN-γ, CXCL9/MIG, CXCL10/IP-10), macrophage activation (IL-6, IL-10, tumor necrosis factor (TNF), CCL2/MCP1, CCL3/MIP-1-α, CCL4/MIP-1β, ferritin), and endothelial injury (vascular endothelial growth factor, VEGF).^25–27^ Exacerbated values of standard laboratory biomarkers such as CRP, ferritin, lymphopenia, and hypoalbuminemia have been linked to ICU admission.^28^ Notably, NT-ProBNP has been proposed as a good predictor of ICU admission in MIS-C.^29^ However, more specific inflammatory biomarkers are needed to better predict severity and guide early treatment.

This study aimed to characterize the inflammatory response during acute MIS-C, before any treatment had been administered, and compare it with convalescent COVID samples at the theoretical MIS-C onset and with longitudinal follow-up samples. We identified a set of inflammatory cytokines and chemokines differentially regulated during acute MIS-C. We also examined ICU-admitted MIS-C patients and identified several biomarkers predictive of more severe outcomes. Finally, we tested the associations of these newly identified biomarkers with classical laboratory biomarkers and the main clinical parameters.

## Methods

### Study approval

This study was approved by the local Ethical Review Board and conducted according to the Declaration of Helsinki. The protocol was approved by the Ethics Committee of Hospital Sant Joan de Déu (PIC-58-60). Pediatric blood samples were taken in Hospital Sant Joan de Déu, Universitat de Barcelona (Spain). Written informed consent was obtained from parents or legal guardians of all participants, and assent was obtained from children when appropriate. Samples of MIS-C were collected before the administration of IVIGs.

### Sex as a biological variable

Our cohort included both male and female participants; therefore, we performed a sex-stratified analysis of the biomarkers differentially regulated in MIS-C. In addition, the analysis of MIS-C severity showed a high proportion of males admitted to the ICU (80%). However, the limited sample size did not provide sufficient statistical power to clearly identify sex differences in each analysis.

### Cohort description. Study design

Samples for this study were collected between March 2020 and February 2022 at the Hospital Sant Joan de Déu, Barcelona, Spain, including distinct groups to provide comprehensive insights into post-infection and disease states.

The convalescent COVID-19 group (CC) contains 17 pediatric individuals. Plasma samples were collected during the convalescent phase, with a median [IQR] of 3.7 [3.1–4.7] weeks post-infection, to analyze the inflammatory profile during convalescence. All individuals in the CC group experienced either asymptomatic or mild acute infection, with no hospitalizations or severe clinical presentations reported.

**Fig. 1:**
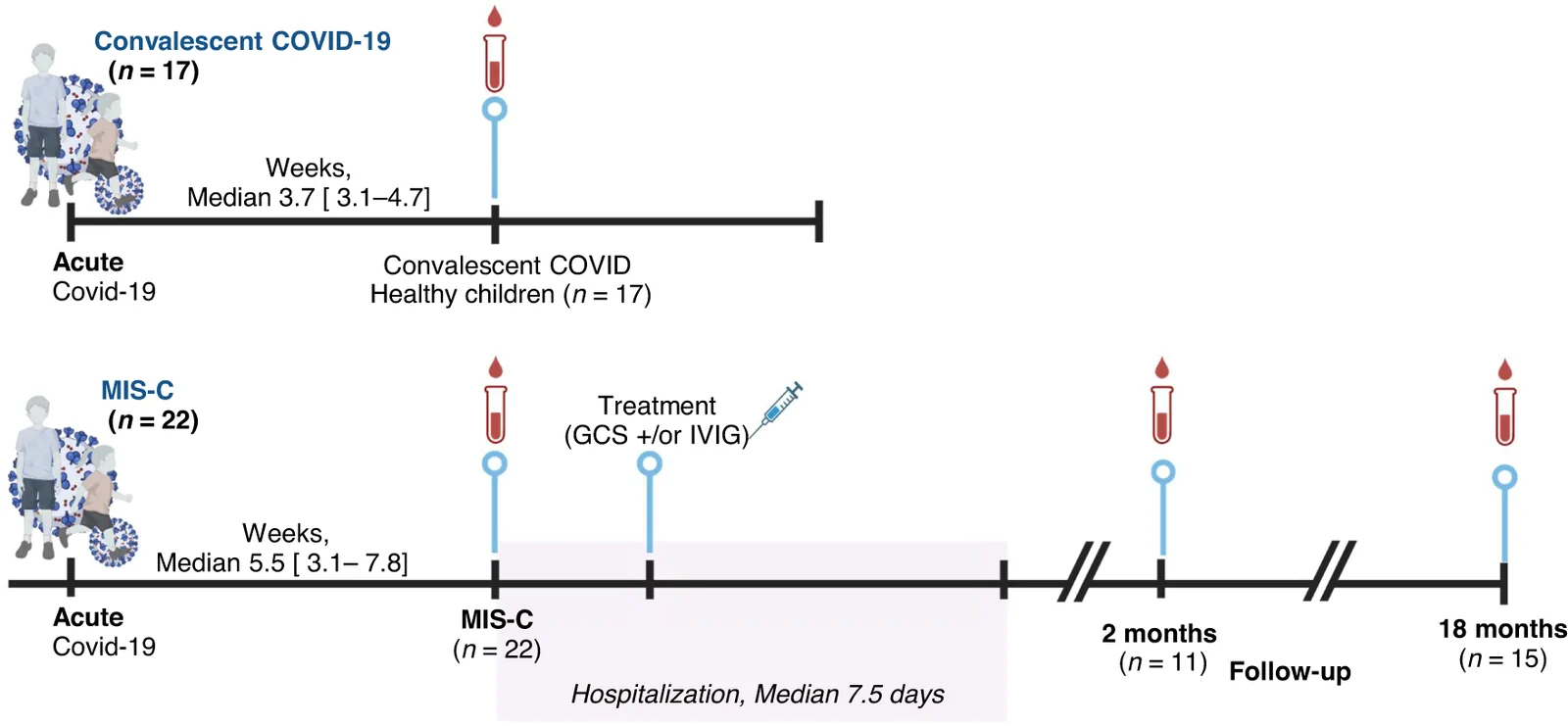
Description of pediatric study cohort: Convalescent COVID-19 and MIS-C groups. Studied groups, including the convalescent COVID-19 group and the MIS-C group. The Convalescent COVID-19 Group (*n* = 17) included children with prior COVID-19, with samples collected 3.7 weeks (IQR: 3.1–4.7 weeks) after diagnosis. The MIS-C group (*n* = 22) included children who developed MIS-C 5.5 weeks (IQR: 3.1–7.8 weeks) after COVID-19, with a median hospital stay of 7.5 days and follow-up samples at 2 months (*n* = 11) and 18 months (*n* = 15).

The MIS-C Group includes 22 pediatric patients diagnosed with MIS-C. MIS-C samples were collected at diagnosis, before any specific treatment (IVIG or corticosteroids), to capture the acute phase of MIS-C. MIS-C diagnosis occurred at a median of 5.5 weeks [3.1–7.8] weeks. Importantly, the calculation of this timeframe is limited by the fact that MIS-C patients were mild or asymptomatic during acute COVID-19 infection, making the exact infection date uncertain. However, the range calculated was in line with other published studies.^3,10,25^ Additionally, this group features a longitudinal component for a subset of the participants, with follow-up samples collected 2 months (*n* = 11) and 18 months (*n* = 15) post-MIS-C diagnosis (Fig. 1).

The clinical diagnostic criteria for MIS-C were established based on the guidelines provided by the WHO,^4^ CDC,^8^, and the Royal College of Pediatrics and Child Health.^17^ Since our participants were diagnosed and samples were collected at the onset of the pandemic, we retained the inclusion of neurological, respiratory, and renal organ system involvement (OSI) in accordance with the original case definitions used during that period.^11^

All MIS-C patients showed substantial recovery during their hospital stay and were released in stable condition.

### Olink proteomics assay

The profile of circulating proteins associated with the inflammatory condition was investigated using the Olink multiplex assay (Olink Proteomics AB, Sweden). Serum samples were analyzed with the Olink Target 96 Inflammation panel, which targets 92 proteins implicated in inflammation. The serum samples were prepared according to the standard protocols recommended by Olink. The multiplex assay was performed following the manufacturer’s instructions, utilizing high-specificity proximity extension assay (PEA) technology. The experiment was performed on a single plate with a single replicate. Data normalization ensured accuracy, with internal controls correcting for within-assay variation and negative controls used to establish the limits of detection. Negative Control, consisting of buffer, is included on each plate in triplicate to monitor any background noise generated when DNA-tags come in close proximity without prior binding to the appropriate protein. The negative controls set the background levels for each protein assay and are used to calculate the limit of detection (LOD) (supplementary dataset). The frequency of missing data, values falling below the LOD, was also recorded for each cytokine (supplementary dataset). The results were reported in NPX units on a Log2 scale, allowing comparative analysis across samples and conditions. The Olink assay was performed at the Immune Response and Biomarkers Core Facility at ISGlobal (Barcelona, Spain).

### Statistics

Volcano plot showing biomarkers differential expression and corresponding unpaired *t* tests was generated with GraphPad Prism (Version 10.4, Graphpad Software, La Jolla, CA).

We used Random Forest to identify biomarkers that best differentiate MIS-C from CC, as well as those associated with severe disease. Feature selection was based on the mean decrease in Gini impurity as the importance score, and biomarkers were selected based on its distribution, retaining those with the highest relevance to the model. Discriminative performance measures of the Random Forest model and the confusion matrix were also extracted. Additionally, stratified analysis by sex was performed to assess potential differences in biomarker behavior across male and female patients.

Group differences based on the selected variables were visualized through heatmaps, and hierarchical clustering was applied to both variables and patients to reveal patterns of similarity. Using GraphPad Prism, the levels of MIS-C patients were compared according to their severity, alongside the CC group, and non-parametric comparison tests (Kruskal-Wallis with Dunn’s multiple comparison test) were performed.

To analyze the longitudinal evolution of the biomarkers, a principal component analysis was performed, highlighting the distinct timepoints to identify whether clusters exist based on these timepoints. Additionally, comparisons were made with GraphPad Prism, using non-parametric tests (Kruskal-Wallis with Dunn’s multiple comparison test) to assess the differences between groups.

To reveal associations between cytokines and clinical markers, a discovery process was performed using linear models for each cytokine and each clinical marker, adjusted for multiple comparisons. Significant associations were then re-evaluated by adjusting for sex and age to account for potential confounding effects.

Protein-Protein Interaction network analysis was performed using the String database (https://string-db.org). Only interactions that were of high confidence (interaction score in STRING database = 0.9) were used.

## Results

### General characteristics of MIS-C and convalescent COVID-19 participants

This study included a total of 22 MIS-C patients and 17 convalescent COVID-19 participants who did not develop MIS-C symptoms. The demographic, clinical, and laboratory characteristics of MIS-C patients and convalescent COVID-19 participants are summarized in Table 1, while the number of patients analyzed is detailed in Fig. 1.

**Table 1:** Demographic, clinical, and laboratory characteristics of MIS-C patients and convalescent COVID-19 participants

	MIS-C (*n* = 22)	Convalescent-COVID-19 (*n* = 17)	*p-*value^A^
**Demographics**			
Age (years), Median [IQR]	7.5 [4.8–9.9]	9.9 [5.8–14.5]	0.0907
Female sex at birth, *n* (%)	10 (45%)	9 (50%)	0.6428
Ethnicity, *n* (%)			
Hispanic Caucasian	10 (45%)	9 (50%)	
Latin American	6 (27%)	8 (44%)	
Romani	3 (14%)	1 (6%)	
North African	2 (9%)		
Eastern Europe	1 (5%)		
**COVID-19**			
Period from COVID-19 diagnosis to sampling (weeks), Median [IQR]	5.5 [3.1–7.8]	3.8 [3.1–4.7]	0.0875
Positive SARS-CoV-2 PCR at sampling, *n* (%)	2 (9%)	0	0.2018
Positive anti-SARS-CoV-2 IgG, *n* (%)	22 (100%)	14 (82%)	0.0403
Positive anti-SARS-CoV-2 IgM, *n* (%)	8 (36%)	5 (28%)	0.6479
COVID-19 vaccination, *n* (%)	3 (14%)	0	0.0728
**MIS-C symptoms & diagnosis**			
Fever duration (days), Median [IQR]	5.0 [4.0–6.0]	n.a.	
Respiratory symptoms, *n* (%)	7 (32%)	n.a.	
Gastrointestinal symptoms, *n* (%)	21 (95%)	n.a.	
Neurologic symptoms, *n* (%)	13 (59%)	n.a.	
Bilateral conjunctivitis, *n* (%)	17 (77%)	n.a.	
Oral/pharynx erythema), *n* (%)	6 (27%)	n.a.	
Peripheral extremity changes, *n* (%)	4 (18%)	n.a.	
Polymorphous rash, *n* (%)	13 (59%)	n.a.	
Lymphadenopathy, *n* (%)	3 (14%)	n.a.	
Cardiological complications, *n* (%)	10 (45%)	n.a.	
Hb (g/dL), Median [IQR]	12 [11–13]	13 [13–14]	0.0003
Platelets (x10^3^/µL), Median [IQR]	115 [103–212]	294 [274–368]	<0.0001
Leukocytes (x10^3^/ µL), Median [IQR]	7.85 [5.5–11.8]	8.1 [6.0–10.2]	0.9088
Lymphocytes (/µL), Median [IQR]	800 [500–1225]	3200 [2100–4200]	<0.0001
Neutrophils (/µL), Median [IQR]	4850 [3850–6875]	3500 [2700–3900]	0.0083
CRP (mg/dL), Median [IQR]	193 [127–241]	0.45 [0.2–1.3]	<0.0001
Procalcitonin (ng/mL), Median [IQR]	3.7 [1.6–11]	0.01 [0.002–0.02]	<0.0001
ALT (U/L), Median [IQR]	33 [17–75]	15 [11–19]	0.0003
AST (U/L), Median [IQR]	39 [31–54]	26 [17–29]	<0.0001
LDG (U/L), Median [IQR]	662 [530–727]	537 [452–635]	0.0156

^A^Mann-Whitney test for continuous variables and Chi square test for percentages.

*IQR* interquartile range, *Hb* hemoglobin, *CRP* C-reactive protein, *ALT* alanine aminotransferase, *AST* aspartate aminotransferase, *LDG* lactate dehydrogenase.

b MIS-C cases were identified 5.5 [3.1–7.8] (median [IQR]) weeks after COVID-19 diagnosis (Fig. 1, Table 1). All MIS-C patients tested positive for SARS-CoV-2 IgG serology, and two (9%) had a positive RT-qPCR test at the time of MIS-C onset. Clinical inflammatory markers were significantly elevated, with all patients showing increased C-reactive protein (CRP) and D-dimer levels. Additionally, 86% had elevated procalcitonin levels, 91% had elevated ferritin levels, and 59% had low platelets. Most MIS-C patients experienced involvement of at least three organ systems. The most affected systems were gastrointestinal (95%), hematological (86%), and mucocutaneous (83%). Cardiovascular involvement was observed in 10 patients (45%, Table 1): 7 of them developed myocarditis, 2 had pericarditis, and 3 were diagnosed with valve dysfunction. Importantly, no cases of coronary artery aneurysms were identified. All cardiological complications developed during the acute phase of MIS-C resolved over time, and by the 18-month follow-up, no alterations of cardiac function were observed. Most patients (21 out of 22, 95%) received a single dose of IVIG. All patients were treated with intravenous corticosteroids and anticoagulation therapy. The median [IQR] hospital stay was 7.5 [5.0–10.0] days.

The MIS-C cohort was divided into two subgroups: the ICU-admitted group (*n* = 12, 55%), who required intensive care due to severe symptoms, and the non-ICU-admitted group (*n* = 10), who were managed in the general inpatient department. Cases requiring ICU admission were characterized by cardiac involvement (highly significant increase in NT-proBNP levels, *p* = 0.0023), hemodynamic instability such as shock (83% of ICU-admitted), exacerbated platelet reduction, and elevated levels of multiple metabolic and inflammatory biomarkers, including LDH, AST, D-dimer, ferritin, and procalcitonin (*p* < 0.05, supplementary Table S1).

Convalescent COVID-19 participants were diagnosed with SARS-CoV-2 infection by RT-qPCR during acute infection, and samples were obtained 3.7 [3.1-4.7] (median [IQR]) weeks after COVID-19 diagnosis. Clinically, at the time of acute SARS-CoV-2 infection, 22% reported respiratory symptoms, 17% gastrointestinal symptoms, and 28% neurological symptoms (headache), all of which were mild. At the time of sample collection and analysis, all convalescent COVID-19 patients tested negative by RT-qPCR. Infection was subsequently confirmed by anti-nucleocapsid protein serology. Blood analyses showed no significant abnormalities.

### Inflammation biomarkers associated with acute MIS-C

**Fig. 2:**
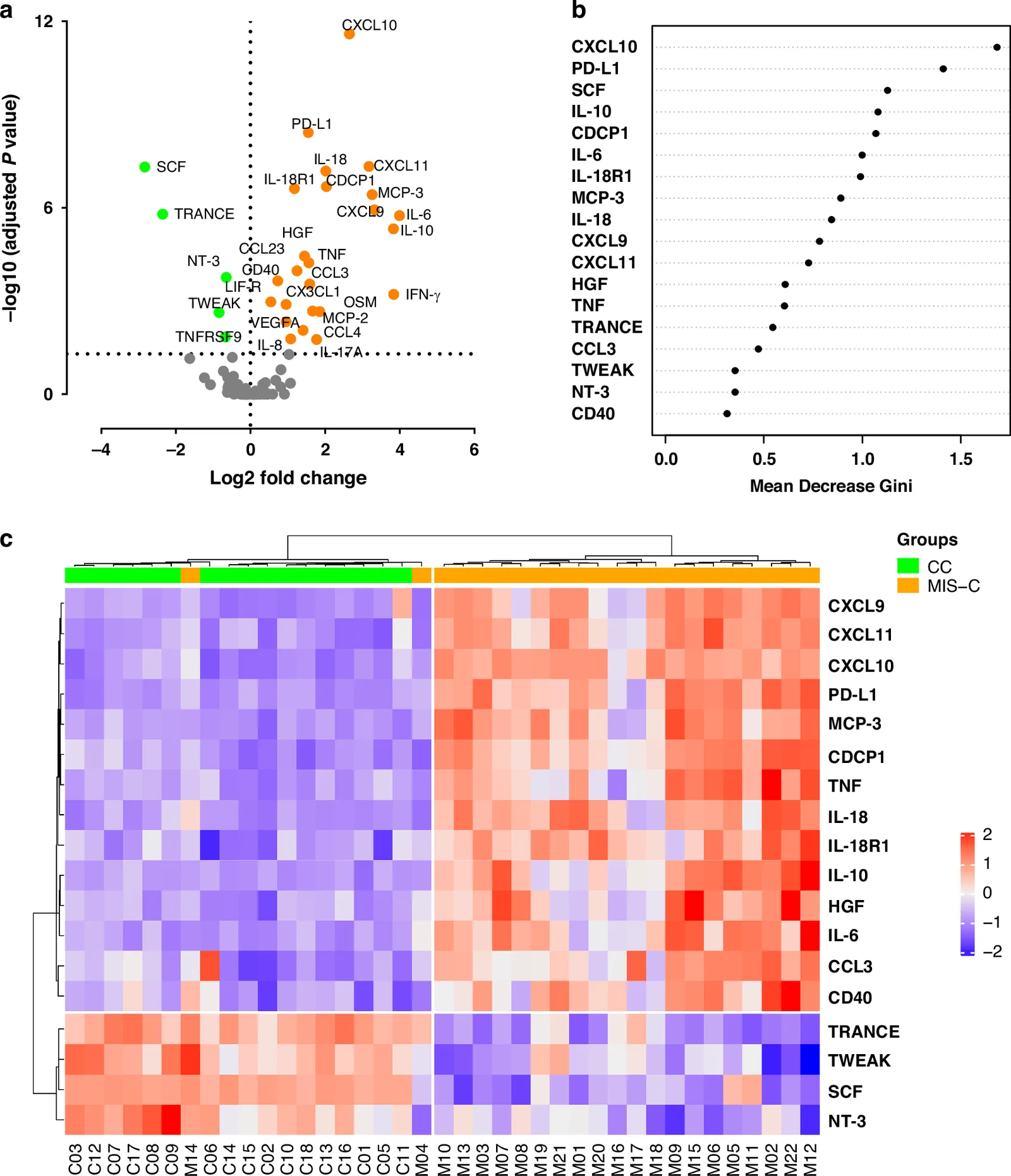
Comparative analysis of inflammatory biomarkers in MIS-C and control COVID groups. We performed an Olink assay to compare soluble biomarkers in convalescent COVID-19 and acute MIS-C individuals. **a** Volcano Plot representation of the relative levels (Log2-fold change) of biomarkers in MIS-C compared to convalescent COVID-19. A horizontal dashed line indicates the threshold to 95% certainty. Protein highlighted in green were significantly upregulated in MIS-C, while proteins highlighted in orange were significantly downregulated. **b** Top 18 cytokines distinguishing MIS-C from the convalescent group extracted from random forest analysis (Fig. S1). **c** Clustered heatmap showing the level pattern of key inflammatory markers selected from Random Forest analysis. Blue indicates low expression levels, while red represents high expression levels.

We first compared the inflammatory response between acute MIS-C, before any treatment, and convalescent COVID-19 participants. Serum samples were analyzed using the T96 Olink technology for 92 inflammatory biomarkers. A direct fold-change analysis identified 29 biomarkers with significant differences between the two groups (Fig. 2a), including 24 showing higher levels in MIS-C and 5 showing higher levels in convalescent COVID-19. The most overexpressed biomarkers in MIS-C were IL-6, IL-10, and IFN-γ, and the most downmodulated were stem cell factor(SCF) and TNF-related activation-induced cytokine (TRANCE). We next performed a Random Forest analysis to narrow down the best differentiating parameters (Fig. 2b, S1). The out of the box (OOB) estimated error rate was 5.13% (Fig. S1). Specifically, all 17 convalescent COVID-19 samples were properly classified, but 2 out of 22 MIS-C cohort patients were misclassified as part of the Convalescent COVID group (Fig. S1). From this analysis, we identified 18 markers considered to have the most influence on the classification of MIS-C and convalescent COVID-19 (Fig. 2b). All these markers were among the 29 identified by the 2 fold-change analysis and included pro-inflammatory cytokines IL-6, TNFα, IL-18 and its receptor IL-18R1, the anti-inflammatory cytokine IL-10, the chemokines CXCL9, CXCL10, CXCL11/I-TAC, CCL3 and MCP3/CCL7, several regulatory molecule such as CD40, CUB domain-containing protein 1 (CDCP1)/CD318, programmed death-ligand 1 (PDL1), TRANCE and TNF-related weak inducer of apoptosis (TWEAK), and the growth factors hepatocyte growth factor (HGF), SCF and neurotrophin-3 (NT3).

Using this set of 18 biomarkers, we performed an unsupervised clustering of acute MIS-C and convalescent COVID-19 samples in which two main groups were identified (Fig. 2c). One group included the majority of the MIS-C individuals (20 out of 22). Most differentiating biomarkers were upregulated in the MIS-C cluster, while TRANCE, TWEAK, SCF, and NT-3 were downregulated. The second group included all convalescent COVID-19 individuals, as well as 2 MIS-C diagnosed individuals who showed a biomarker profile indistinguishable from the convalescent COVID-19 group.

To further investigate the functional relationships among the 29 selected biomarkers from the volcano plot, we conducted a functional protein–protein interaction analysis using the STRING database (Fig. S2), which revealed the involvement of TNF, IL-10 and INF-γ pathways in the pathophysiology of MIS-C.

Sex stratified random forest analyses identified separate sets of biomarkers differentiating MIS-C vs. convalescent COVID-19 within male and female participants, with only 9 markers common to both analyses: IL-10, CDCP1, CXCL10, CXCL11, IL-18, PD-L1, MCP-3, SCF, and IL-6 (Fig. S3a, b). Notably, the classification of female participants between convalescent COVID-19 and MIS-C was less effective (Fig. S3c) and included the two MIS-C samples misclassified as convalescent COVID-19 in random forest and clustering analyses.

### Inflammation biomarkers associated with ICU admission in MIS-C

Next, we analyzed inflammatory responses between the more severe MIS-C cases requiring ICU care and the less severe cases managed in the general inpatient unit. This grouping strategy was expected to identify biomarkers that could predict the requirement for ICU admission. Main characteristics of these subgroups are shown in Supplementary Table S1, identifying a high percentage of males in the ICU group.

**Fig. 3:**
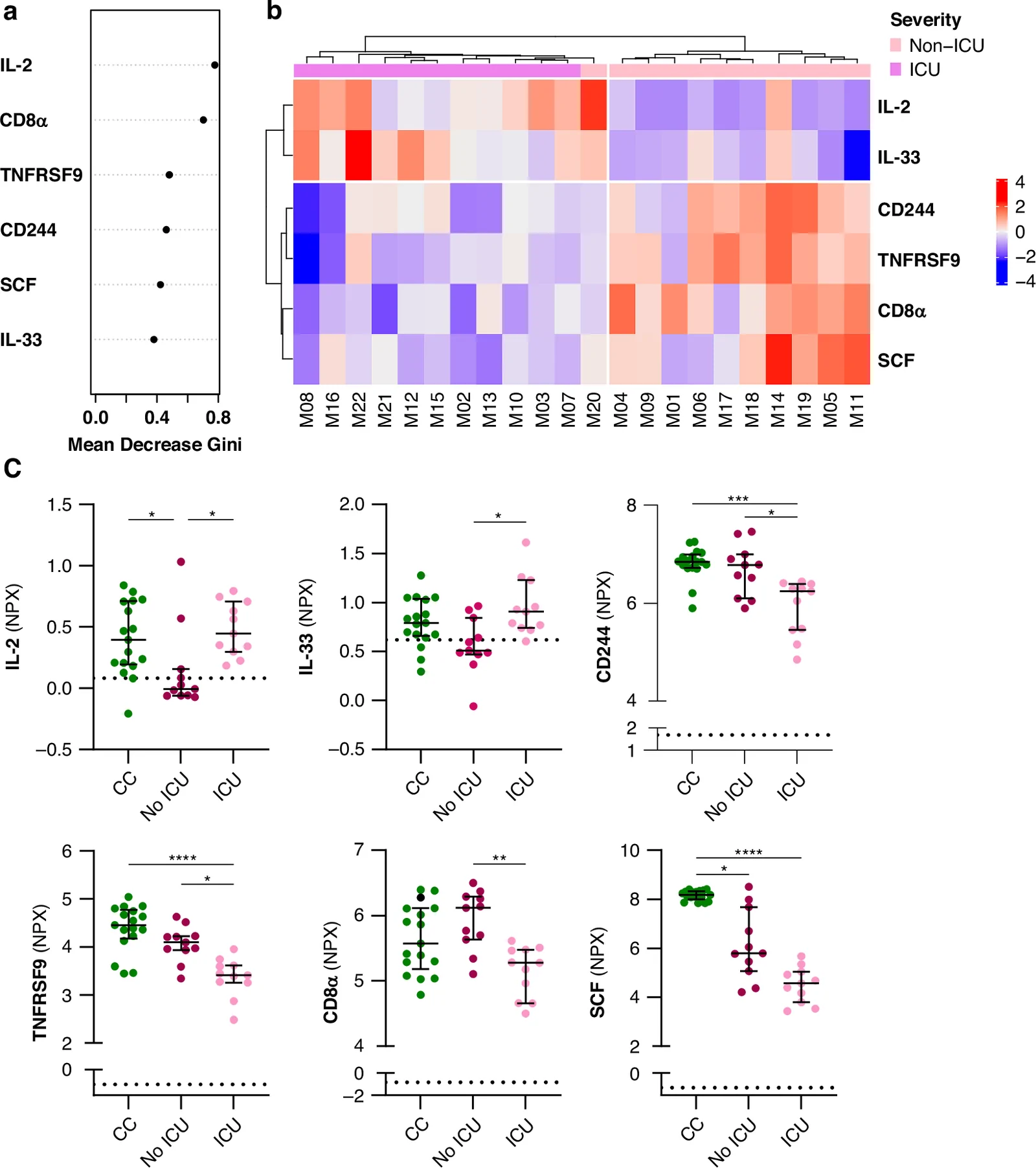
Inflammatory biomarkers signature of MIS-C ICU-admission. **a** Top 6 biomarkers Identified by Random Forest Analysis (Fig. S4) discriminating MIS-C cases with ICU admission vs. non-ICU. **b** Clustering - Heatmap of Cytokine levels differentiating ICU- and non-ICU admitted MIS-C cases based on the most significant inflammatory markers. **c** Top 6 biomarkers individual levels comparing convalescent COVID group, ICU, and non-ICU MIS-C cases. *P* values were analyzed using Kruskal-Wallis test, with significance levels indicated as follows: *P ≤ 0.05, **P ≤ 0.01, ***P ≤ 0.001, ****P ≤ 0.0001. Horizontal bars indicate median values, error bars show interquartile ranges, and dashed lines indicate LODs.

Following the same strategy described above, we identified by random forest analysis the inflammatory markers best capable of segregating ICU-admitted and non-ICU-admitted MIS-C cases (Fig. S4). Among the 6 best biomarkers, two were upregulated in severe cases (IL-2 and IL-33), and four were downregulated in severe cases (soluble CD8α, TNF-receptor superfamily 9 (TNFRSF9), CD244 and SCF, Fig. 3a). Applying these 6 parameters in an unsupervised clustering of the MIS-C participants allowed for the separation of all patients except for one non-ICU MIS-C whose biomarker profile resembled the one of ICU participants (Fig. 3b). When comparing individually these markers in convalescent COVID-19, MIS-C non-ICU and MIS-C ICU, we observed three main situations. TNFRSF9 and SCF both showed a trend for downregulation within MIS-C non-ICU vs. convalescents and lower levels in MIS-C ICU vs. non-ICU. CD244 was significantly decreased in MIS-C ICU compared to CC (*p* = 0.0004) and MIS-C non-ICU (*p* = 0.0169), but was similar in convalescent and MIS-C non-ICU groups. IL-33 and soluble CD8α both showed differential levels between MIS-C non-ICU and MIS-C ICU (*p* = 0.0160, *p* = 0.0024, respectively) but no significant difference between MIS-C groups and convalescent COVID-19. IL-2 showed the most surprising pattern with a strong downregulation in MIS-C non-ICU compared to both CC (*p* = 0.0404) and MIS-C ICU (*p* = 0.0113), but no difference between CC and MIS-C ICU. Importantly, IL-2 and IL-33 exhibited a high frequency of missing data (21% and 33%, respectively) with very low concentrations in the non-ICU MIS-C group, in which the majority of measurements fell below the limit of detection (LOD; 0.08288 and 0.61705 NPX, respectively).

### Co-associations between inflammatory biomarkers and clinical parameters in MIS-C

**Fig. 4:**
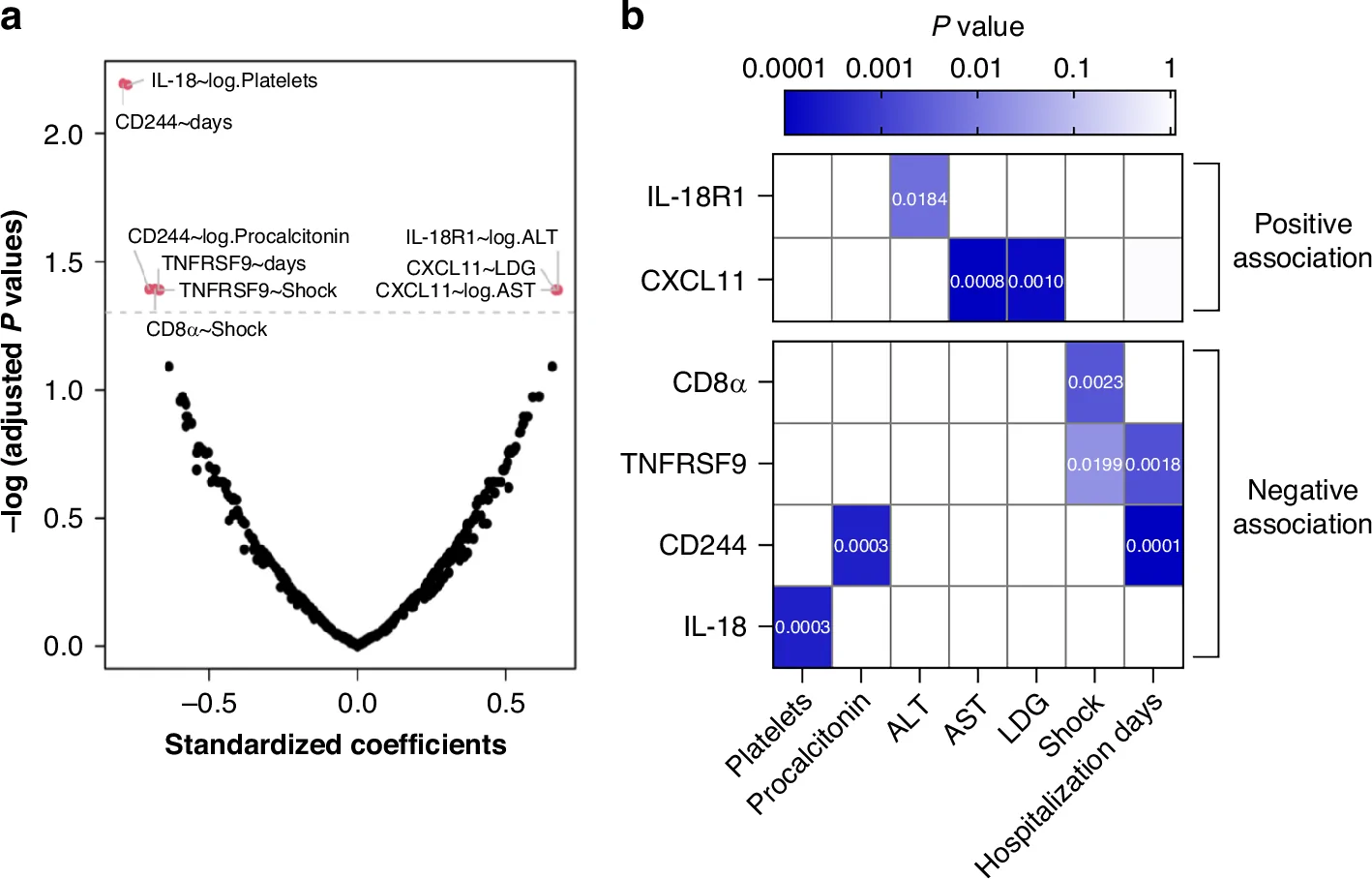
Co-associations between biomarkers and clinical parameters. **a** The plot shows the standardized coefficients from regression analyses, allowing for comparison across different biomarkers and clinical parameters. The y-axis represents the negative log-transformed adjusted *p*-values (-log10), indicating the significance of associations. Higher values suggest stronger statistical significance, with key biomarkers indicated. Positive coefficients indicating a positive association and negative coefficients indicating a negative association. The horizontal dashed line marks the significance threshold corresponding to an adjusted *p*-value of 0.05. **b** A table representing the significant associations between biomarkers and clinical parameters, using linear regression models, adjusting by age and sex. The color gradient represents the *p*-values, with darker blue indicating stronger significance in the association. *P*-values are only indicated for significant associations. The upper section of the heatmap includes biomarkers positively associated with clinical parameters (IL-18R1 and CXCL11), while the lower section includes negatively associated biomarkers (CD8A, TNFRSF9, CD244, and IL-18).

We used linear regression models to evaluate the association between the key biomarkers identified above and routine clinical parameters assessed for diagnosis within the MIS-C group. Figure 4a shows the standardized obtained coefficients and their p-values corrected for multiple testing using FDR method. Significant associations discovered were re-evaluated, adjusting for age and sex, to verify that the effect was not confounded by these variables (Fig. 4b). We observed an association between lower CD244 levels and both elevated procalcitonin levels (*p* = 0.0003) and longer hospitalization duration (*p* = 0.0001). Shock was associated with the downregulation of CD8α (*p* = 0.0023) and TNFRSF9 (*p* = 0.0199), with TNFRSF9 also associated with prolonged hospitalization (*p* = 0.0018). In addition, increased CXCL11 levels were associated with high AST (*p* = 0.0008) and LDH levels (*p* = 0.0010). Finally, we also identified a relationship between platelet loss and increased IL-18 (*p* = 0.003) and a positive link between IL-18R1 and ALT levels (*p* = 0.0184), further highlighting the complex interplay between inflammation, organ function, and disease severity in MIS-C.

### Longitudinal Stabilization of Biomarker Profiles Post-MIS-C Treatment

**Fig. 5:**
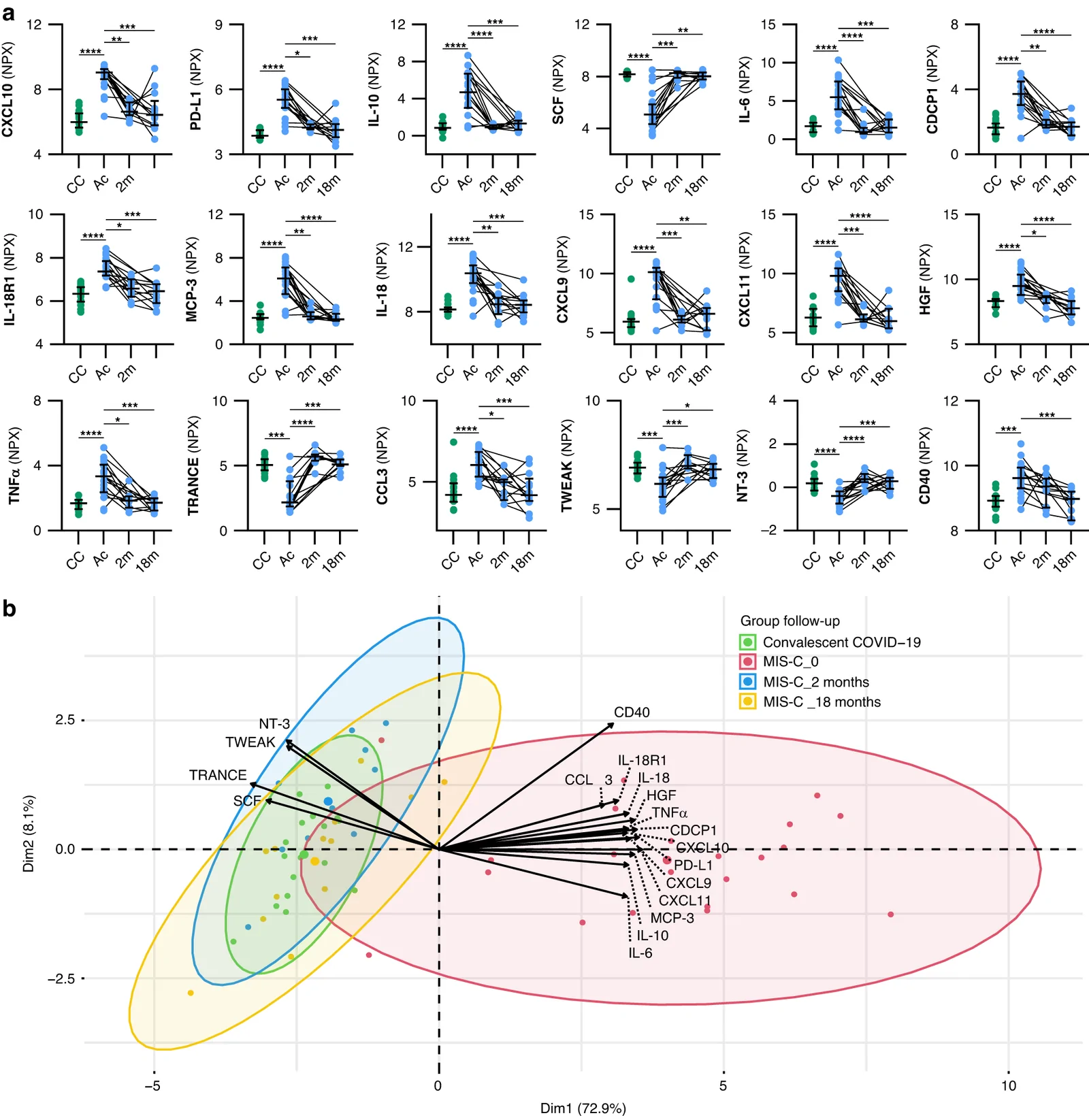
Biomarkers stabilization post-MIS-C treatment. **a** Biomarker stabilization over time: Individual biomarkers dot plots showing their levels in convalescent COVID-19 and across MIS-C follow-up, at acute disease, 2-month later, and 18-month later. Differences between groups and timepoints were analyzed using the Kruskal-Wallis test, with significance values indicated as follows: * *P* ≤ 0.05, **P ≤ 0.01, ***P ≤ 0.001, ****P ≤ 0.0001. Horizontal bars indicate median values and error bars show Interquartile ranges. **b** Principal component analysis: Representation of acute MIS-C, 2-month and 18- month MIS-C follow-ups, and convalescent COVID-19 using the first two components of the analysis on soluble biomarkers.

Some of the MIS-C participants provided blood samples 2 and/or 18 months after acute disease (Fig. 1). Our longitudinal analysis of biomarker levels post-treatment for MIS-C revealed a normalization of the 18 biomarkers previously identified by 2 months after the disease onset that was sustained until 18 months (Fig. 5a). Principal Component Analysis shown in Fig. 5b, using the same set of biomarkers, suggested that the inflammatory marker landscape in the 2-month and 18-month follow-up timepoints were undistinguishable from that of the convalescent COVID-19 group, in stark contrast to the distinct profile observed during acute MIS-C. This convergence suggests a rapid and full immunological recovery over the extended follow-up period.

## Discussion

In the present study, we analyzed 92 inflammation-related soluble biomarkers comparing acute MIS-C and convalescent COVID-19 children using the Olink technology. We identified 24 markers significantly upregulated in MIS-C and 5 significantly downmodulated when compared to convalescent COVID-19. Upregulated biomarkers, including CXCL10, CXCL9, CXCL11, MCP3, IL-6, IL-10, TNF, CCL3, PD-L1, CD40, HGF, IL-18, and CDCP1, are in line with previous reports25,26,^30–34^ comparing acute MIS-C with healthy controls and using different technique,s including Olink, Meso Scale Discovery, Proximity Extension Assay, and cytometric bead-based multiplex assay panels.

Using a Random Forest analysis, we narrowed down a set of 18 markers capable of differentiating convalescent COVID-19 and MIS-C individuals. Among the selected biomarkers, CXCL11 showed a significant correlation with liver damage markers. Several studies have highlighted CXCL10 and CXCL11 as potential biomarkers for acute liver injury.^35,36^ Furthermore, the combined elevation of CXCL9, CXCL10, and CXCL11 with the elevation of NT-pro BNP has been associated with cardiac injury,^37,38^ suggesting a broader impact on organ systems. Notably, CXCL10 is well-established as a key chemokine in the immunopathology of SARS-CoV-2 in adults,^39^ and our findings suggest a similar pathogenesis may be present in children with MIS-C. Upregulation of the proinflammatory cytokine IL-18 has been well documented in MIS-C.^31,34^ Here, we detected a strong association between increased IL-18 and low platelet levels. Platelets are a main source of IL-18 in blood, which they release upon activation^40^, and IL-18 blood levels have been correlated with thrombocytopenia in sepsis.^41^ Our data suggest that IL-18 could also be a good surrogate marker for platelet activation in MIS-C. We also observed the downregulation of SCF, TRANCE, TWEAK, and TNFRSF9 in MIS-C. TRANCE and TNFRSF9 downmodulation have been reported by Reiter et al.^31^ Sacco et al also identified SCF downmodulation as a classifying marker of MIS-C compared to acute COVID-19.^26^ Finally, and consistent with previous studies, we identified high expression of IFN-γ in MIS-C patients.^25,26,42^ However, it was only ranked highly in the female-specific random forest analysis and was more variable in males. On the other hand, IFN-γ downstream chemokine CXCL9 was retained as a key biomarker when considering all participants. In this regard, there is a clinical and immunological overlap between MIS-C and Macrophage activation syndrome (MAS), sharing persistent fever, multiorgan involvement, and hyperinflammatory markers such as elevated CRP, ferritin, AST, D-dimers with low platelets, and cytokines including IL-6, IL-18, and TNF-α.^23,42,43^ Both MIS-C and MAS also show elevated levels of IFN-γ and CXCL9, suggesting that both the interferon pathway and macrophage-driven immune responses are involved in these diseases.^33,44^ In our study, formal diagnostic criteria for Hemophagocytic Lymphohistiocytosis (HLH) or MAS in sJIA were not applied to exclude MAS. However, although several patients showed elevated ferritin levels (>684 ng/mL), none of them met sufficient key criteria, including multiple cytopenia or documented hemophagocytosis, suggesting that MAS was not present in our cohort. ^45^.

Using Random Forest analysis, we identified 6 biomarkers: IL-2, IL-33, CD244, soluble TNFRSF9, soluble CD8α, and SCF, with the capacity to classify MIS-C individuals admitted or not to ICU. Interestingly, these molecules are all immunomodulatory proteins and are notably involved in T cells and NK cells development and activation. One male participant, M20, was misclassified as an ICU-admitted MIS-C patient but did not develop severe complications. The only differentiating parameters of M20 were their ethnicity (Eastern Europe) and the fact that they had received a complete COVID-19 vaccination one month prior to MIS-C diagnosis. However, in our cohort, two additional children were vaccinated prior to MIS-C onset, including a 13-year-old child fully vaccinated 5 months before MIS-C who was admitted to the ICU. Despite this, a study showed a reduction of MIS-C cases in vaccinated children and suggested a reduction of severe presentations.^46^ Soluble TNFRSF9 (also called CD137 or 4-1BB) was significantly reduced in all MIS-C compared to convalescent COVID-19 participants, and it was identified in the random forest analysis as a key marker differentiating between MIS-C ICU and non-ICU. It also showed a significant negative association with shock and hospital stay. Altogether, these results suggest that the plasma level of soluble TNFRSF9 could be a valuable biomarker for the early prediction of MIS-C outcomes. Reiter et al. also reported soluble TNFRSF9 downmodulation in MIS-C^31^ and proposed a link with myocarditis. We noticed an association of soluble TNFRSF9 downmodulation with increased myocardial dysfunction in univariate analyses however, this association was lost after applying age and sex adjustment. In general, soluble TNFRSF9 association with shock and ICU admission was more robust. Soluble CD8α downmodulation also showed good association with shock. Low levels of both soluble TNFRSF9^47^ and CD8α^48^ are associated with reduced CD8 + T cell activation. On the other hand, soluble TNFRSF9 and CD8α can also have immunosuppressive activity,^49,50^, and their downmodulation could be related to the stronger inflammatory response in the MIS-C cases. Either way, this finding underscores the potential role of CD8 + T cell dysregulation in the pathophysiology of severe, ICU-admitted, MIS-C. Interestingly, soluble TNFRSF9 has been shown to be increased in ICU-admitted severe COVID-19 cases, suggesting a different role than in MIS-C.^51^ In contrast, it is worth noting that in our cohort, neither IFN-γ nor CXCL9 was a good biomarker to predict ICU-associated MIS-C cases. IL-2 and IL-33 both showed an unexpected pattern. IL-33 appeared to be upregulated in ICU-admitted patients, consistent with prior reports linking it to severity,^52,53^, but interpretation is limited by the low sensitivity of these 2 cytokines and a strong association with male sex, which may act as a confounder. Indeed, we noticed a strong association between IL-33 levels and male sex in both MIS-C and convalescent COVID-19, a pattern that has been reported in prior research.^54^ IL-2 levels were reduced in non-ICU MIS-C despite previous studies reporting elevation in severe cases.^55^ Given that both cytokines frequently fell below the limit of detection and showed substantial missingness, they should not be considered reliable diagnostic or prognostic markers for MIS-C severity. Future studies with higher-sensitivity assays are needed to clarify the potential biological role of IL-2 and IL-33 in MIS-C. Finally, the downregulation of CD244 in ICU-admitted patients was associated with prolonged hospital stays and elevated procalcitonin levels, suggesting that it may be a good biomarker for more severe conditions in MIS-C development. Procalcitonin, a well-established biomarker of inflammation, is particularly elevated in bacterial infections and is widely used to differentiate between bacterial and viral causes of inflammation. Notably, previous studies have reported that CD244 levels are reduced in bacterial infections and MIS-C, as what was observed in our study.^56^ This parallelism between CD244 downregulation and elevated procalcitonin levels highlights a potential shared immune dysregulation pathway in MIS-C and bacterial infections, which warrants further investigation.

Sex stratified random forest analysis suggested some differences between male and female participants; however, the size of the cohort did not allow for a deeper analysis. In addition, we observed a strong unbalance of males associated with ICU admission (9/12, 75%) compared to females (3/8, 27%). Statistics for male sex in ICU-MIS-C at the level of the Catalan region were 69%.^3^ Many studies have reported a higher proportion of males in MIS-C cases^57^, but there is no clear association between being male and increased severe outcomes.^12^ Among the female participants, two MIS-C, M04 and M14, exhibited very divergent biomarker profiles and were misclassified as convalescent COVID-19 in our clustering analysis. Both patients were Hispano-Caucasian, without comorbidities, and had 5 days of fever with the shortest hospital stay (4 days) among all the MIS-C participants. M14 met laboratory criteria for MIS-C, presenting with hematologic, neurological, gastrointestinal, and mucocutaneous involvement. M04 was the least severe MIS-C case, with only gastrointestinal and hematologic involvement, and did not receive IVIG due to the absence of severe symptoms and reassuring clinical blood analytics, including stable cardiac markers and no signs of hemodynamic instability. While the definition of MIS-C has evolved since 2020 and was formerly revised in 2023^58^ there is still an overlap between MIS-C cases and other inflammatory diseases, notably Kawasaki disease.^11^

A relevant contribution from our study is the longitudinal analysis of inflammatory biomarkers. Although MIS-C patients have been clinically followed in different studies,^19,20,59^, limited information exists on the behavior of inflammatory biomarkers. Our data shows a complete normalization of all up-regulated and down-regulated biomarkers to the levels present in convalescent COVID-19 as soon as 2 months after diagnosis and treatment. An observation that was further confirmed in the long term (18 months).

## Conclusions

This study provides a refined characterization of the MIS-C-associated inflammatory response, identifying key biomarkers that could differentiate MIS-C from convalescent COVID-19 and predict disease severity among the MIS-C cases. Importantly, anti-inflammatory treatment was effective in normalizing all biomarkers in the short and long term (2 and 18 months, respectively). These insights may improve early detection, guide clinical management, and inform future research on post-viral inflammatory syndromes.

### Limitations

Our study is limited by the small cohort size and the single-center design, which limits generalizability. As we chose to compare acute MIS-C with convalescent COVID-19, our study does not allow us to specifically distinguish MIS-C from severe COVID-19-associated inflammatory responses. Sex distribution within ICU-admitted and non-admitted MIS-C was not balanced, which could affect our results. The longitudinal study did not allow us to distinguish the individual effects of IVIG and corticosteroid treatments on cytokine dynamics. Finally, a key limitation of our study was the lack of functional immune assays, such as T cell activation or intracellular cytokine staining, which limits the mechanistic interpretation of the identified biomarkers. Future studies incorporating such assays will be essential to validate and expand upon our findings.

## Supplementary information


Supplementary Material
Dataset


## Data Availability

The raw Olink NPX dataset generated during the study is available as supplementary material.
