# Supplementary material for: Tracking the soluble biomarkers of MIS-C and their association with clinical parameters and severity
Source: Pediatr Res. 2025 Oct 31;100(1):420–30. doi: 10.1038/s41390-025-04542-8 (PMC13524822; doi:10.1038/s41390-025-04542-8)
Supplement: Supplementary file 1 [file 41390_2025_4542_MOESM1_ESM.pdf]

Supplementary table S1. Comparison of clinical features and inflammatory biomarkers between ICU- and non-ICU-admitted MIS-C patients.

	ICU-admitted (n=12)	Non-ICU-admitted (n=10)	*p*-value[A]
**Demographics**			
Age (years), Median [IQR]	7.8 [6.9-12.05]	6.1 [3.5-9.8]	0.1970
Female sex at birth, n (%)	3 (25%)	7 (70%)	0.0348
**MIS-C symptoms & diagnosis**[B]			
OSI hematological, n (%)	12 (100%)	7 (70%)	0.0412
OSI mucocutaneous, n (%)	9 (75%)	9(90%)	0.1052
OSI musculoeskeletal, n (%)	0	0	
OSI cardiac, n (%)	8 (67%)	2 (20%)	0.0286
OSI neurological, n (%)	8 (67%)	5 (50%)	0.4285
OSI respiratory, n (%)	4 (33%)	3 (30%)	>0.9999
OSI renal, n (%)	0	0	
OSI gastrointestinal, n (%)	12 (100%)	9 (90%)	0.2622
Number of OSIs (total), Median [IQR]	4.5 [4-5]	3 [3-4]	0.0167
Shock, n (%)	11 (92%)	0	<0.0001
**Treatment and outcomes**			
IVIG treatment	12	9 (90%)	0.2622
IVIG repeated course	0	0	
Corticosteroids	12 (100%)	10 (100%)	
Anticoagulation therapy	12 (100%)	10 (100%)	
Stay in hospital, days	9.5 [6.3 - 10.0]	5.5 [4.0 - 8.3]	0.0272
**Laboratory data**			
Hb(g/dL), Median [IQR]	11.8 [10.2-12.9]	12 [11.3-12.7]	0.8085
Platelets (x10^3/ µL), Median [IQR]	108 [96-138]	207 [108-262]	0.0358
Leukocytes (x10^3/µL), Median [IQR]	7.15 [4.825-13.65]	8.15 [6.35-9.475]	0.7846
Lymphocytes (/µL), Median [IQR]	800 [325-1150]	1050 [500-2175]	0.2076
Neutrophils (/µL), Median [IQR]	4100 [3100-9100]	5350 [4250-6800]	0.5711
CRP (mg/dL), Median [IQR]	223 [148-251]	151 [107-229]	0.1072
D-Dimer (ng/ml), Median [IQR]	5850 [2393-18633]	3520 [2310-4405]	0.1932
Procalcitonin (ng/mL), Median [IQR]	7.5 [2.9-41.5]	2.3 [0.24-4.5]	0.0249
Ferritin (microg/L), Median [IQR]	941 [379-1204]	376 [232-590]	0.0345
NT-proBNP (ng/L), Median [IQR]	6556 [3583-12317]	263 [203-3011]	0.0023
Troponin I (ng/ml), Median [IQR]	0.018 [0.01-0.84]	0.008 [0.001-0.023]	0.0736
ALT (U/L), Median [IQR]	39 [23-95]	21 [14-43]	0.0716
AST (U/L), Median [IQR]	44 [39-93]	34 [26-39]	0.0237
LDG (U/L), Median [IQR]	700 [628-758]	566 [475-680]	0.0358
Albumin (g/dL), Median [IQR]	4.5 [4-5]	3.8 [3-4]	0.0517

[A] Mann-Whitney test for continuous variables and Chi square test for percentages.

[B] Organ System Involvement (OSI) was defined from the following specific symptoms: Hematological (platelets <150,000/µL or ALC <1,000/µL), Mucocutaneous (rash, conjunctivitis, oral changes, or hand erythema/edema), Musculoskeletal (myalgia, arthralgia, or myositis), Cardiac (LVEF <55%, coronary changes, or elevated troponin), Neurologic (altered mental status, headache, weakness, or paraesthesia), Respiratory (need for oxygen, lower respiratory infection, chest x-ray infiltrates, effusion, bronchospasm, or haemorrhage), Renal (acute kidney injury or renal replacement therapy), Gastrointestinal (abdominal pain, vomiting, or diarrhea). IQR, interquartile range; Hb, hemoglobin; CRP, C-reactive protein; ALT, alanine aminotransferase; AST, aspartate aminotransferase; LDG, lactate dehydrogenase.

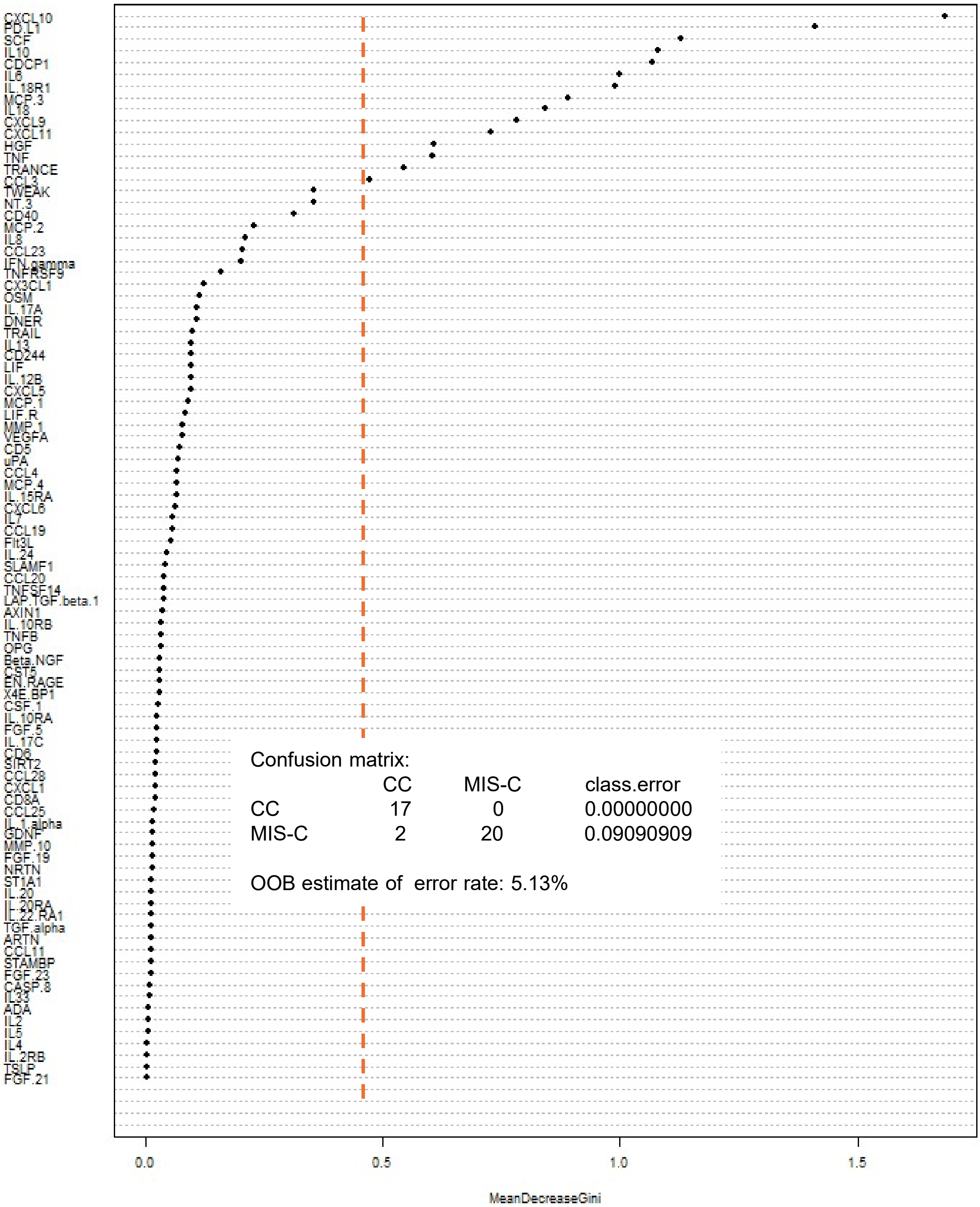

Figure S1. Random forest analysis between convalescent COVID-19 and acute MIS-C. Ranking of soluble biomarkers differentiating convalescent COVID-19 and acute MIS-C according to the Random Forest analysis **(corresponds to Figure 2)**.

MIS-C vs. Convalescent COVID-19

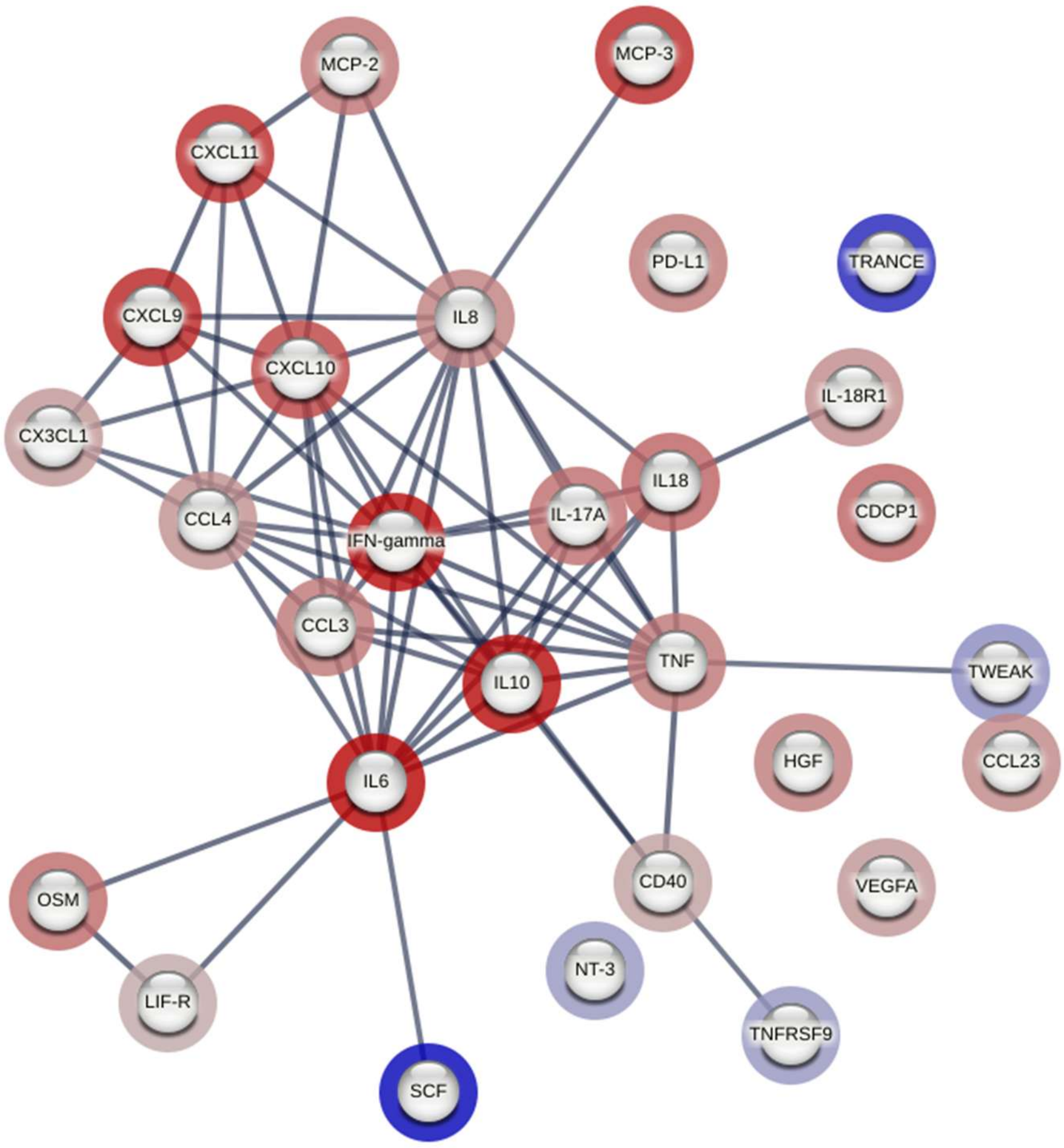

Figure S2. Protein-protein Interaction Network Based on STRING Analysis. STRING network analysis visualizing the protein-protein interactions among differentially expressed biomarkers. The classification is based on volcano test (**Fig.2A**). Proteins shown in red are upregulated in MIS-C group, while blue nodes represent downregulated proteins. The highest positive values for MIS-C were IL-6 (3.988), IFN-γ (3.834), IL-10 (3.829), with lowest values of SCF (-2.831) and TRANCE (-2.355).

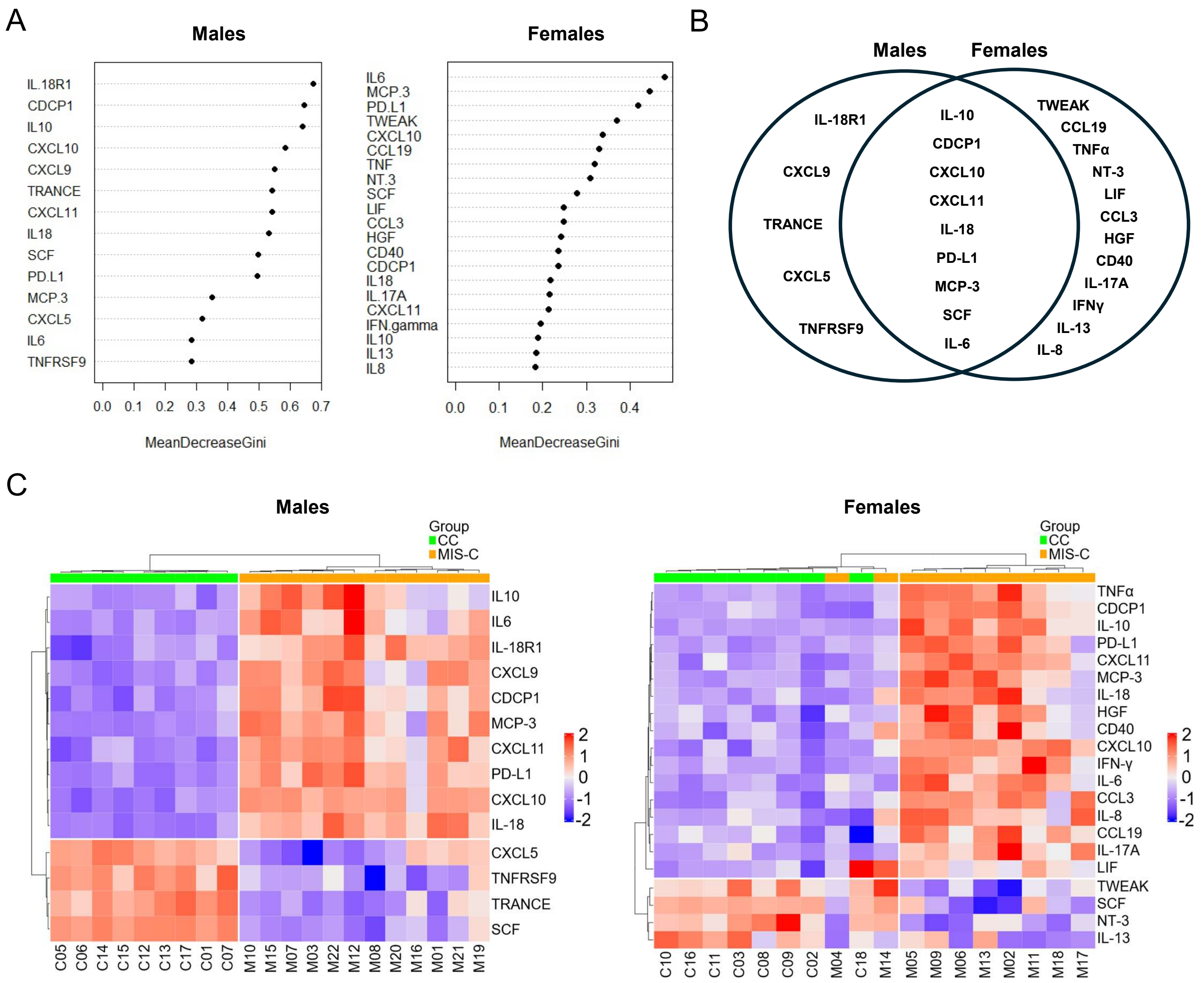

Figure S3. Sex stratified random forest analysis between convalescent COVID-19 and acute MIS-C. (A). Top cytokines identified by random forest analysis in each sex group. The random forest analysis highlights the most important cytokines distinguishing convalescent COVID-19 and acute MIS-C for males (left panel) and females (right panel). **(B). Venn Diagram of sex-specific and shared cytokines.** The Venn diagram compares cytokines identified by random forest analysis in males and females. Shared cytokines (center) are involved in both groups, while sex-specific cytokines (left for males, right for females) indicate potential sex-based immune response differences. **(C). Heatmaps of cytokine levels.** Heatmaps illustrate the levels patterns of cytokines selected by random forest analysis in males (left) and females (right). Red indicates higher levels, while blue represents lower levels, showing distinct profiles.

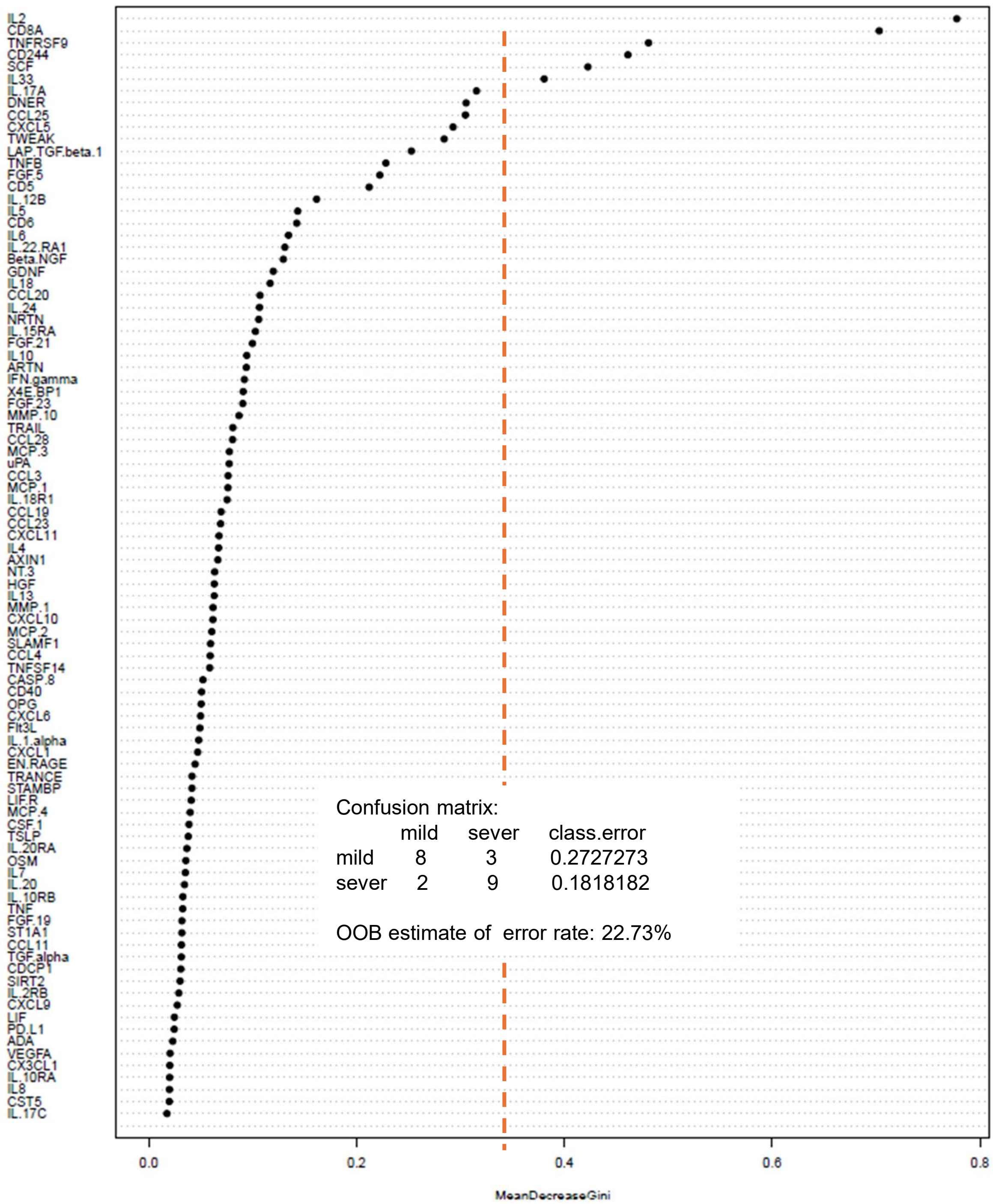

Figure S4. Random forest analysis between ICU-admitted and non-ICU-admitted MIS-C patients. A random forest-based ranking was conducted to identify the key soluble inflammatory markers measured by Olink that best distinguish between severe MIS-C cases requiring ICU care and milder cases treated in the general inpatient unit **(see Figure 3)**.
